# An exploration of the role of a fish-oriented diet in cognitive decline: a systematic review of the literature

**DOI:** 10.18632/oncotarget.16347

**Published:** 2017-03-17

**Authors:** Ling-Feng Zeng, Ye Cao, Wei-Xiong Liang, Wen-Hu Bao, Jian-Ke Pan, Qi Wang, Jun Liu, Hao-Dong Liang, Hui Xie, Yan-Ting Chai, Zi-Tong Guan, Qian Cao, Xiao-Yan Li, Lei Yang, Wei-Hua Xu, Sui-Qing Mi, Ning-Sheng Wang

**Affiliations:** ^1^ Institute of Clinical Pharmacology, Guangzhou University of Chinese Medicine, Guangzhou, China; ^2^ The 2nd Affiliated Hospital of Guangzhou University of Chinese Medicine, Guangdong Provincial Hospital of Chinese Medicine, Guangzhou, China; ^3^ Department of Clinical Research/National Clinical Trials Institute, Sun Yat-sen University Cancer Center, Guangzhou, China; ^4^ World Federation of Chinese Medicine Societies, Beijing, China

**Keywords:** cognitive disorders, dementia of Alzheimer type, fish-oriented dietary intake, risk factors, meta-analysis

## Abstract

Epidemiological studies have presented inconsistent evidence of the correlation between a fish-oriented dietary intake (FDI) and the risk of cognitive decline. To address these controversies, we performed this systematic review of prospective studies published in December 2016 and earlier using PubMed, Embase, and Web of Science. Two independent researchers conducted the eligibility assessment and data extraction; all discrepancies were solved by discussion with a third researcher. The pooled relative risks (RRs) focused on the incidence of events were estimated with 95% confidence intervals (CIs). Overall, nine studies containing 28,754 subjects were analyzed. When the highest and lowest categories of fish consumption were compared, the summary RR for dementia of Alzheimer type (DAT) was 0.80 (95%CI = 0.65–0.97); i.e., people with a higher intake of fish had a 20% (95%CI = 3–35%) decreased risk of DAT. Additionally, the dose-response synthesized data indicated that a 100-g/week increase in fish intake reduced the risk of DAT by an additional 12% (RR = 0.88, 95%CI = 0.79–0.99). Non-significant results were observed for the risk of dementia of all causes (DAC) and mild cognitive impairment (MCI). Limited evidence involving heterogeneity was found within subgroups or across studies. In conclusion, this review confirmed that a higher intake of fish could be correlated with a reduced risk of DAT. Further research, especially prospective studies that specifically quantify FDI, will help find a more accurate assessment of the different levels of dietary intake.

## INTRODUCTION

As the population ages worldwide, it is expected that the prevalence of cognitive disorders, including dementia of Alzheimer type (DAT), mild cognitive impairment (MCI), and dementia of other causes, might increase in future decades [1–2]. Thus, it is of enormous significance to further clarify the potential risk factors (e.g., individual diet) [3]. Regarding the role of nutrition in the prevention of cognitive disorders, increased hope has focused on a fish-oriented dietary intake (FDI), since fish is an important source of omega-3 fatty acids (n-3 FAs) [4–5]. In particular, recent studies found that n-3 FAs, including docosahexaenoic acid (DHA), were present in the membranes of brain tissue [6]. Eicosapentaenoic acid (EPA) also functions as a protective factor in the nervous system of human subjects [7]. Furthermore, other studies reported that a diet enriched with DHA could lessen the neurodegenerative pathology and protect from cognitive decline in aged rats [8–9].

Recently, a substantial body of evidence has supported the hypothesis that the regular consumption of an FDI may decrease the risk of cardiovascular morbidity [10] and mortality [11–13], stroke [14], and other age-related disorders. However, the evidence from observational literature reveals an inconsistent association between FDI and the risk of cognitive decline. Some studies demonstrated that a higher FDI was associated with a lower risk of cognitive disorders [15–17], whereas others failed to prove such protective correlations [18–19]. One possible reason for these conflicting results is the limited capacity of dietary recall surveys and food frequency questionnaires to quantify fatty acid levels.

Given the uncertain evidence regarding the roles of an FDI and the risk of cognitive decline, we performed this updated systematic review by searching and analyzing published studies. In addition, this meta-analysis was limited to prospective cohort studies because case-controlled studies might contain bias, especially when it comes to recalling previous dietary habits after cognitive disorders have been diagnosed. Also, it was assumed that the heterogeneity among results might be much smaller when similar study designs are considered.

## RESULTS

### Results of the search

**Figure 1:**
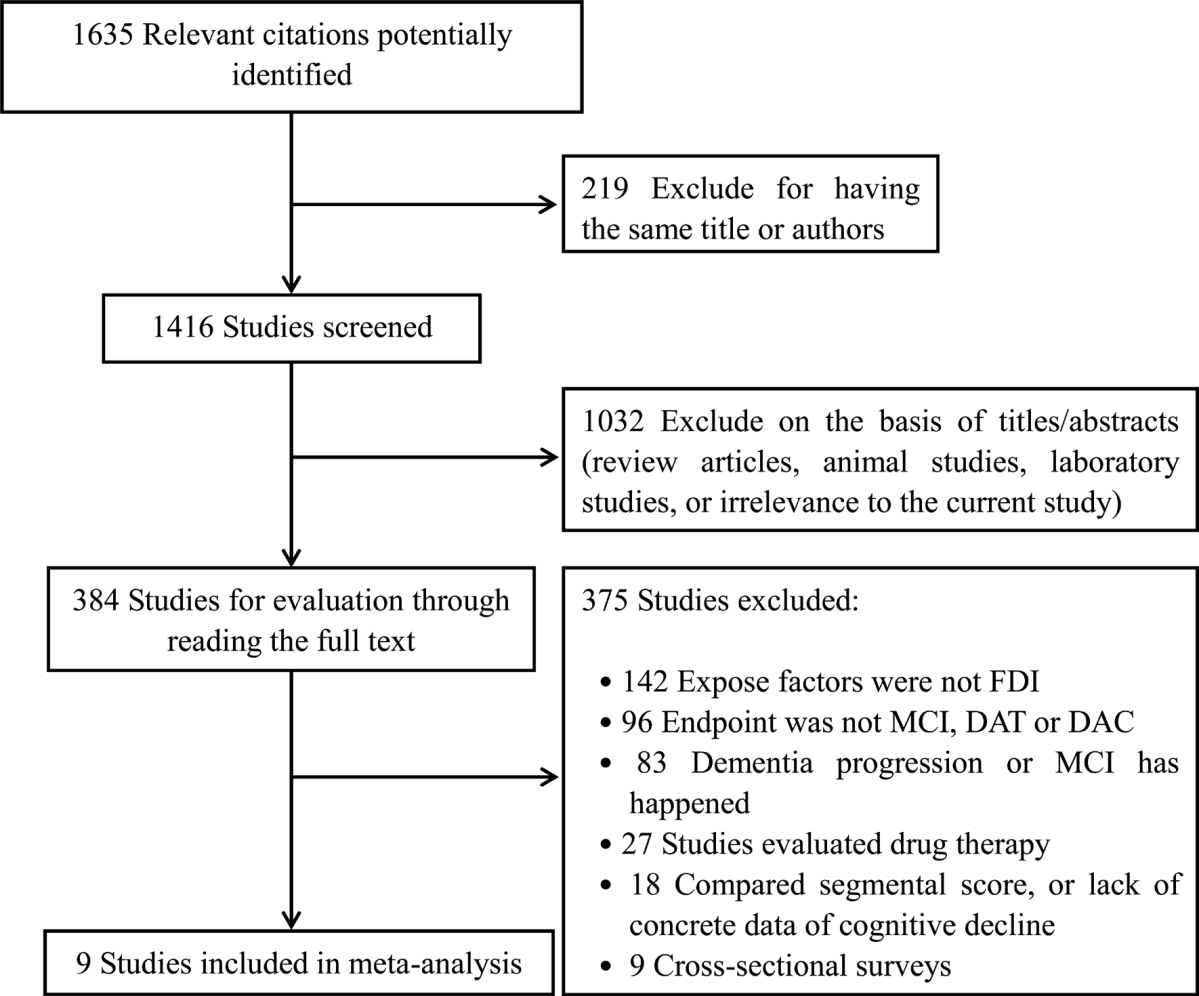
Flow diagram of the trial-selection process DAT = dementia of Alzheimer type; DAC = dementia of all causes; MCI = mild cognitive impairment; FDI = fish-oriented dietary intake.

The initial retrieval yielded 1635 publications. Of these, 1251 articles were excluded for the following reasons: 219 included duplicate authors or titles; 1032 contained unmatched content (e.g. animal studies, reviews, laboratory articles, or other irrelevant topics). Of the remaining 384 studies, 142 did not mention the characteristics of the FDI (including fish, n-3 FAs supplementation, or other fish-related products.), 96 did not report the endpoint of DAT, MCI, or DAC, 83 included patients in whom dementia had progressed or MCI occurred, 27 focused on assessing drug therapies, and 18 compared segmental dementia scores as outcome indices instead of performing complete data measurements. Despite attempts to contact the authors *via* telephone or e-mail, we did not receive replies to allow access to the data for the above 18 studies. In addition, nine studies recruited subjects in cross-sectional surveys. Finally, nine eligible studies [15–23] containing a total of 28,754 subjects were included for further analysis (Figure 1).

### Characteristics at baseline and quality assessment

The quality of the nine prospective cohort studies was assessed using the Newcastle-Ottawa Scale (NOS) [24] are the results are displayed in Table 1. The evaluated points ranged from 6-7, with overall quality judgments of “moderate to inferior”. The characteristics of the included studies are presented in Table 2. The age of all study subjects was ≥55 years. The incidence of DAT ranged from 0.69-16.1%, DAC was 1.08-16.9%, and MCI was 13.2-23.9%. The different patterns of FDI were documented separately (such as the intake of fish, n-3 FAs, DHA, or EPA).

**Table 1:** Methodological quality of the prospective cohort studies by using the Newcastle-Ottawa Scale^#^

First author, year	Selection				Comparability^§^	Exposure or outcome			No. of star
	1	2	3	4		1	2^†^	3^‡^	
Morris MC, 2003	*	*	*	*	* *	*			7
Schaefer EJ, 2006	*	*	*	*	* *		*		7
Kalmijn S, 1997	*	*	*	*	*		*		6
Barberger-Gateau P, 2007	*	*	*	*	*		*	*	7
Olsson E, 2015	*	*	*	*	*		*	*	7
Roberts RO, 2010	*	*	*	*	*		*		6
Huang TL, 2005	*	*	*	*	*		*		6
Chan R, 2013	*	*	*	*	*		*	*	7
Devore EE 2009	*	*	*	*	* *		*	*	8

**^#^The Newcastle-Ottawa Scale criteria are listed in supplemental files.** A study could be awarded a maximum of one star for each item except for the item “Comparability”. §A maximum of 2 stars could be awarded for this item. Studies that controlled for total energy intake received one star, whereas studies that controlled for other important confounders such as body mass index received an additional star. †A cohort study with a follow-up time > 10 y was assigned one star. ‡A cohort study with a follow-up rate > 75% was assigned one star.

**Table 2:** Characteristics of the cohort studies included in the meta-analysis

First author, year, sources	Study population	Subjects (n)		Age(yrs), mean/range	Male, >n (%)	Types of FDI	Estimates of FDI	No. of events, n (%)	Methods for event detection	Length of follow-up (yrs), mean/range	Maximum adjustment available
		Baseline	Follow-up								
Morris MC, 2003, CHAP	United State	3352	815	NP/65-94	318 (39.1%)	Fish, n-3 FAs, DHA, EPA	FFQ	DAT, 131 (16.1%)	NINCDS-ADRDA, NE	3.9/NP	Age, sex, race, education, total energy intake, APOE-∈4 status (any ∈4 vs. none), the interaction between race and APOE-∈4, and period of observation;
Schaefer EJ, 2006, FHS	United State	1921	899	76/55-88	328 (36.5%)	Fish, DHA	SFFQ	-DAT, 71 (7.89%) -DAC, 99 (11.1%)	DSM-IV, MMSE, NINCDS-ADRDA, NE	9.1/NP	Age, sex, APOE ∈4 allele, plasma homocysteine concentration, education level, energy intake, BMI, hypertension, diabetes mellitus, smoking status, alcohol intake, and history of stroke;
Kalmijn S, 1997, RDS	Netherlands	7983	5386	68/≥55	2204 (40.9%)	Fish	SFFQ	-DAT, 37 (0.69%) -DAC, 58 (1.08%)	CAMDEX, MMSE, NINCDS-ADRDA, GMS, DSM-III-R, NE	2.1/NP	Age, sex, education, total energy intake, cigarette smoking, alcohol consumption, fiber consumption, and antioxidant intake;
Barberger-Gateau P, 2007, 3CS	France	9079	8085	NR/≥65	3169 (39.2%)	Fish	FFQ	-DAT, 183 (2.26%) -DAC, 281 (3.48%)	NINCDS-ADRDA, DSM-IV, NE	3.48/2-4	Age, gender, education, city, income, and marital status, APOE genotype, BMI, smoking, hypertension, hypercholesterolemia, and diabetes;
Olsson E, 2015, ULSAM	Sweden	1138	1038	71/NP	1038 (100%)	Fish	FFQ	-DAT, 84 (8.09%) -DAC, 143 (13.8%)	NINCDS-ADRDA, DSM-IV, MMSE, NE	9.8/NP	Age, education, physical activity level, single, household, APOE genotype, total energy intake, high total cholesterol, BMI, smoking, hypertension, diabetes;
Roberts RO, 2010, MCSA	United State	1969	1233	80.1/70-89	641 (51.9%)	Fish, n-3 FAs	FFQ	MCI, 163 (13.2%)	DSM-IV, CDR, NE	2.7/2.5-3.7	Age, sex, education, total energy intake, diabetes, stroke, ApoE ∈4 allele status, coronary heart disease, BMI, depressive symptoms, marital status, dyslipidemia, alcohol intake, cigarette smoking, and moderate exercise;
Huang TL, 2005, CHCS	United State	5201	2233	72/≥65	927 (41.5%)	Fish	FFQ	-DAT, 190 (8.51%) -DAC, 378 (16.9%)	NINCDS-ADRDA, DSM-IV, MMSE, NE	5.4/0.1–8.4	Age at baseline, education, race, gender, minority status, most recent income, presence of APOE ∈4, energy intake, baseline BMI and region;
Chan R, 2013, CSCHK	China	4000	3670	72.2/≥65	1926 (52.5%)	Fish	FFQ	MCI, 877 (23.9%)	CSI-D, NE	3/NP	Age, BMI, PASE, total energy intake, education, Hong Kong ladder, community ladder, smoking habit, alcohol use, no. of ADLs, GDS category, history of diabetes, hypertension, cardiovascular disease or stroke;
Devore EE 2009, RDS	Netherlands	7046	5395	68/≥55	2210 (40.9%)	Fish, n-3 FAs, DHA, EPA	SFFQ	-DAT, 168 (3.11%)^a^ -DAT, 197 (3.65%) ^b^ -DAC, 465 (8.62%)	CAMDEX, MMSE, NINCDS-ADRDA, GMS, DSM-III-R, NE	9.6/1-14	Age, sex, education, total energy intake, alcohol intake, smoking, BMI, high total cholesterol, hypertension, dietary intake of vitamin E, supplement use (either fish, omega-3, or antioxidant supplements), and history of stroke, myocardial infarction, or type 2 diabetes mellitus.

Annotation: CHAP = Chicago Health and Aging Project; FHS = Framingham Heart Study; RDS = Rotterdam Study; 3CS= Three-City Study; ULSAM = Uppsala Longitudinal Study of Adult Men; MCSA = Mayo Clinic Study of Aging; CHCS = Cardiovascular Health Cognition Study; CSCHK = Community Study for Cognition in Hong Kong; NINCDS-ADRDA = National Institute of Neurological and Communicative Diseases and Stroke-Alzheimer Disease and Related Disorders Association; DSM-IV = Diagnostic and Statistical Manual of Mental Disorders (4th Edition); DSM-III-R = Diagnostic and Statistical Manual of Mental Disorders (3th Edition, Revised); CSI-D = Community Screening Instrument for Dementia; CDR = Clinical Dementia Rating Scale; GMS = Geriatric Mental State schedule; MMSE = Mini-Mental State Examination; NE = neurological exam; ADL = Activities of Daily Living; GDS = Geriatric Depression Scale; PASE = Physical Activity Scale for the Elderly; BMI = body mass index; SFFQ = Semi-quantitative Food Frequency Questionnaire; FFQ = Food Frequency Questionnaire; DHA = docosahexaenoic acid; EPA = eicosapentaenoic acid; n-3 FAs = total long-chain omega-3 fatty acids; APOE = Apolipoprotein E; DAT = dementia of Alzheimer type; DAC = dementia of all causes; MCI = mild cognitive impairment; NP = not provided.

^a^ The result was based on 0–8 years' follow-up; ^b^ The result was based on 9-14 years' follow-up.

### Quantitative data synthesis

#### Meta-analysis on fish intake and the risk of DAT

**Figure 2:**
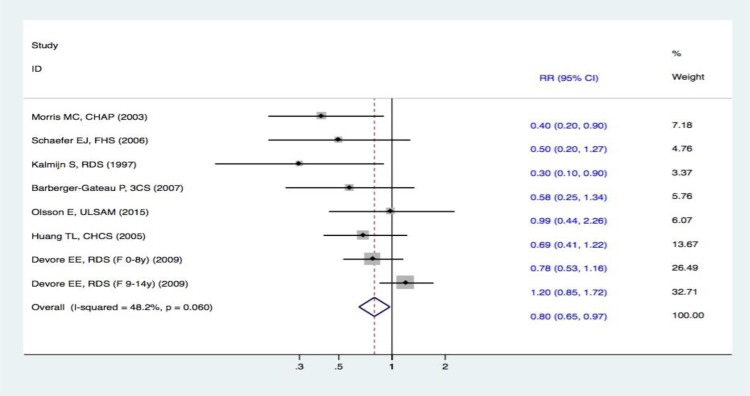
Forest plots (fixed effect model) of meta-analysis on fish intake and risk of DAT Squares indicate study-specific risk estimates (size of the square reflects the study-specific statistical weight); horizontal lines indicate 95% CIs; diamond indicates the summary relative risk with its 95% CI. RR = relative risk; CI = confidence interval; DAT = dementia of Alzheimer type.

Seven studies [15–21] reported the incidence of DAT. The fixed effects meta-analysis revealed a significant difference between subjects in the highest to lowest categories of fish intake. The RR for DAT between the two groups was (RR, 0.80 [95% CI, 0.65-0.97]) (Figure 2). These results indicated that subjects with a higher intake of fish had a 20% (95% CI, 3-35%) decreased risk of DAT. The pooled effect estimates for fish intake were toward a lower risk of DAT.

#### Meta-analysis on fish intake and the risk of DAC

**Figure 3:**
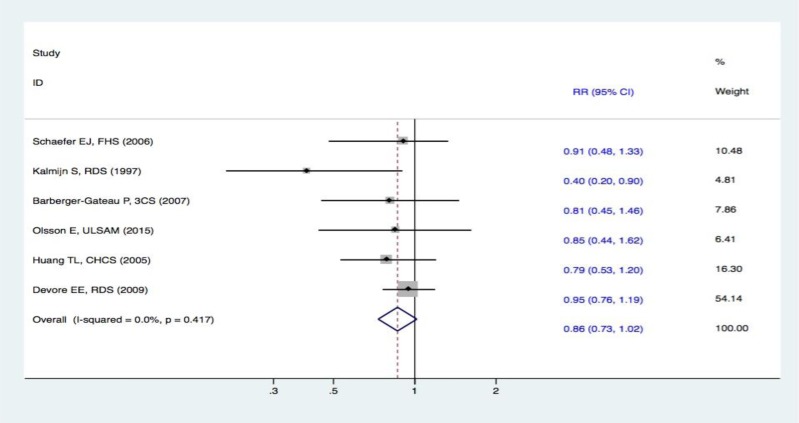
Forest plots (fixed effect model) of meta-analysis on fish intake and risk of DAC Squares indicate study-specific risk estimates (size of the square reflects the study-specific statistical weight); horizontal lines indicate 95% CIs; diamond indicates the summary relative risk with its 95% CI. RR = relative risk; CI = confidence interval; DAC = dementia of all causes.

Six studies [16–21] evaluated the correlation between fish consumption and the risk of DAC. One study [16] revealed that fish intake reduced the risk of DAC, whereas the other five studies [17–21] failed to demonstrate such a relationship. Overall, the synthesized evidence for the risk of DAC with a fixed-effect displayed that there were no statistically significant differences in subjects in the highest and lowest categories of fish intake (RR, 0.86 [95% CI, 0.73-1.02]) (Figure 3). The results revealed that fish intake was not correlated with a decreased risk of DAC.

#### Meta-analysis on fish intake and the risk of MCI

**Figure 4:**
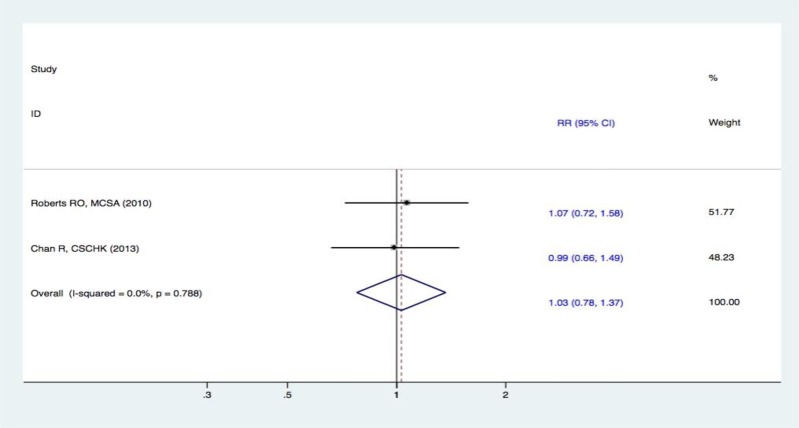
Forest plots (fixed effect model) of meta-analysis on fish intake and risk of MCI Squares indicate study-specific risk estimates (size of the square reflects the study-specific statistical weight); horizontal lines indicate 95% CIs; diamond indicates the summary relative risk with its 95% CI. RR = relative risk; CI = confidence interval; MCI = mild cognitive impairment.

Two studies [22–23] reported the association between fish intake and the risk of MCI. Compared with the lowest intake category, there was no statistically significant association in the pooled analysis for the highest category of fish intake and the risk of MCI (RR, 1.03 [95% CI, 0.78-1.37]) (Figure 4).

### Publication bias

**Figure 5:**
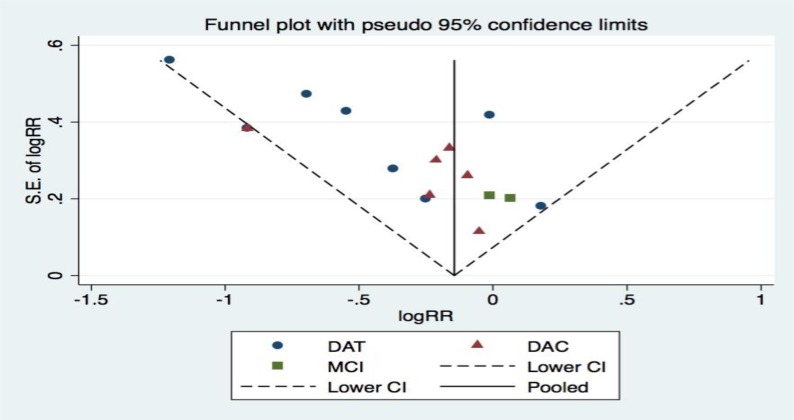
Publication bias for the studies included in the meta-analysis Funnel plot of meta-analysis on fish intake and risk of cognitive decline. RR = relative risk; S.E. = standard error. In the absence of publication bias, the points should be symmetrical about the vertical line at the pooled RRs. The reasonably symmetrical distribution suggests the absence of publication bias.

Publication bias was assessed using funnel plots and RRs constructed from studies involving fish intake and the risk of cognitive decline. In the absence of publication bias, the points should be symmetrical around the vertical line involving the pooled RRs. The shape of the funnel plot appeared to be reasonably symmetrical, which suggested the absence of publication bias (Figure 5).

### Meta-analysis on the intake of n-3 FAs and the risk of DAT, DAC, and MCI

**Figure 6:**
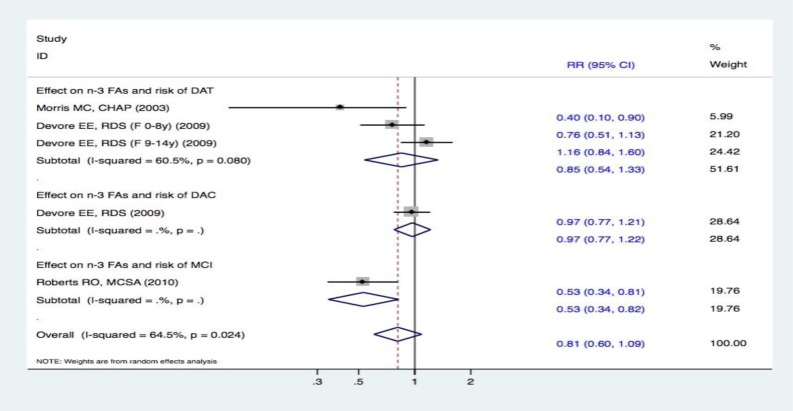
Forest plots (random effect model) of meta-analysis on omega-3 fatty acid intake and risk of events: A. DAT; B. DAC; C. MCI Squares indicate study-specific risk estimates (size of the square reflects the study-specific statistical weight); horizontal lines indicate 95% CIs; diamond indicates the summary relative risk with its 95% CI. RR = relative risk; CI = confidence interval; DAT = dementia of Alzheimer type; DAC = dementia of all causes; MCI = mild cognitive impairment; n-3 FAs = total long-chain omega-3 fatty acids.

Two studies [15, 19] evaluated the intake of n-3 FAs and the incidence of DAT. When comparing the highest and lowest intake categories, one study [15] reported that n-3 FAs could decrease the risk of DAT; however, the other [19] failed to demonstrate such a relationship. Overall, the meta-analysis using a random effects model revealed that there were no statistically significant differences between the two groups (RR, 0.85 [95% CI, 0.54-1.33]). Furthermore, one study [15] reported the association between n-3 FA intake and the risk of DAC. Compared with the lowest category of n-3 FA intake, there was no statistically significant correlation between the highest category and risk of DAC (RR, 0.97 [95% CI, 0.77-1.22]). Additionally, one study [19] focused on the association between the intake of n-3 FAs and the risk of MCI. A higher intake of n-3 FAs was associated with a lower risk of MCI (RR, 0.53 [95% CI, 0.34-0.82]). However, the overall effects of pooled analyses involving the intake of n-3 FAs and the risk of cognitive decline did not display any significant difference between the two groups (RR, 0.81 [95% CI, 0.60-1.09]) (Figure 6).

### Meta-analysis on DHA intake and the risk of DAT and DAC

**Figure 7:**
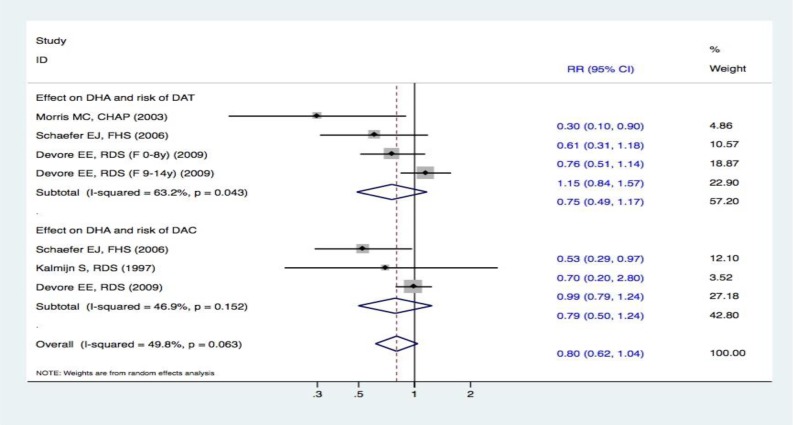
Forest plots (random effect model) of meta-analysis on DHA intake and risk of events: A. DAT; B. DAC Squares indicate study-specific risk estimates (size of the square reflects the study-specific statistical weight); horizontal lines indicate 95% CIs; diamond indicates the summary relative risk with its 95% CI. RR = relative risk; CI = confidence interval; DAT = dementia of Alzheimer type; DAC = dementia of all causes; DHA = docosahexaenoic acid.

Three studies [15, 19, 20] reported DHA intake and the risk of DAT. One study [15] revealed that DHA intake probably reduced the risk of DAT, whereas the others [19, 20] failed to identify such an association. Overall, the pooled effect estimates using a random-effects meta-analysis showed that there was no statistically significant difference between subjects in the highest and lowest categories of DHA intake and the risk of DAT (RR, 0.75 [95% CI, 0.49-1.17]). Moreover, three studies [16, 19, 20] assessed the correlation between DHA intake and the risk of DAC. Compared with the lowest category of DHA intake, there was no statistically significant association between the highest category and the risk of DAC (RR, 0.79 [95% CI, 0.50-1.24]). Overall, the RR between the two groups of DHA intake and the risk of cognitive declines was 0.80 (95% CI, 0.62-1.04). Therefore, there were no obvious effects in the pooled results between DHA intake and the risk of cognitive decline (Figure 7).

### Meta-analysis on EPA intake and the risk of DAT and DAC

**Figure 8:**
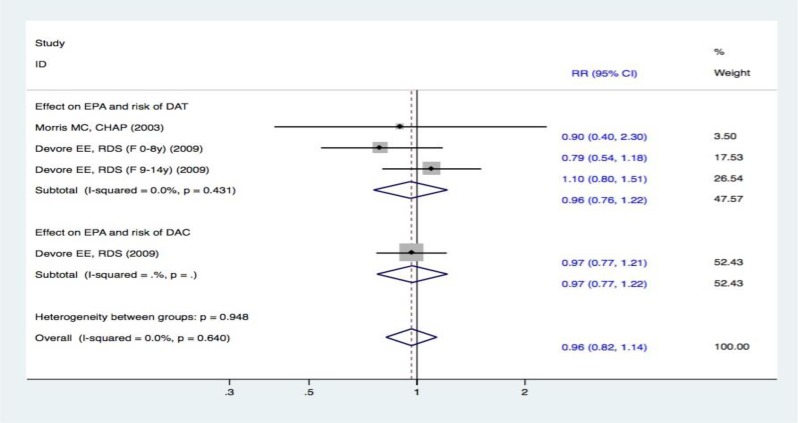
Forest plots (fixed effect model) of meta-analysis on EPA intake and risk of events: A. DAT; B. DAC Squares indicate study-specific risk estimates (size of the square reflects the study-specific statistical weight); horizontal lines indicate 95% CIs; diamond indicates the summary relative risk with its 95% CI. RR = relative risk; CI = confidence interval; DAT = dementia of Alzheimer type; DAC = dementia of all causes; EPA = eicosapentaenoic acid.

Two studies [15, 19] reported EPA intake and the risk of DAT. The fixed effect meta-analysis revealed that there was no significant difference between subjects in the highest and lowest categories of EPA intake. The RR in the incidence of DAT between the two groups was 0.96 (95% CI, 0.76-1.22). These results demonstrate that the pooled effect for EPA intake was not connected to the risk of DAT. Furthermore, one study [19] analyzed EPA intake and the risk of DAC. The results showed no definite correlation between EPA intake and the risk of DAC (RR, 0.97 [95% CI, 0.77-1.22]). Overall, no positive effects were observed in the pooled analysis of EPA intake and the risk of cognitive decline (RR, 0.96 [95% CI, 0.82-1.14]) (Figure 8).

### Subgroup analyses of the correlation between fish intake and the risk of DAT, DAC, and MCI

**Figure 9:**
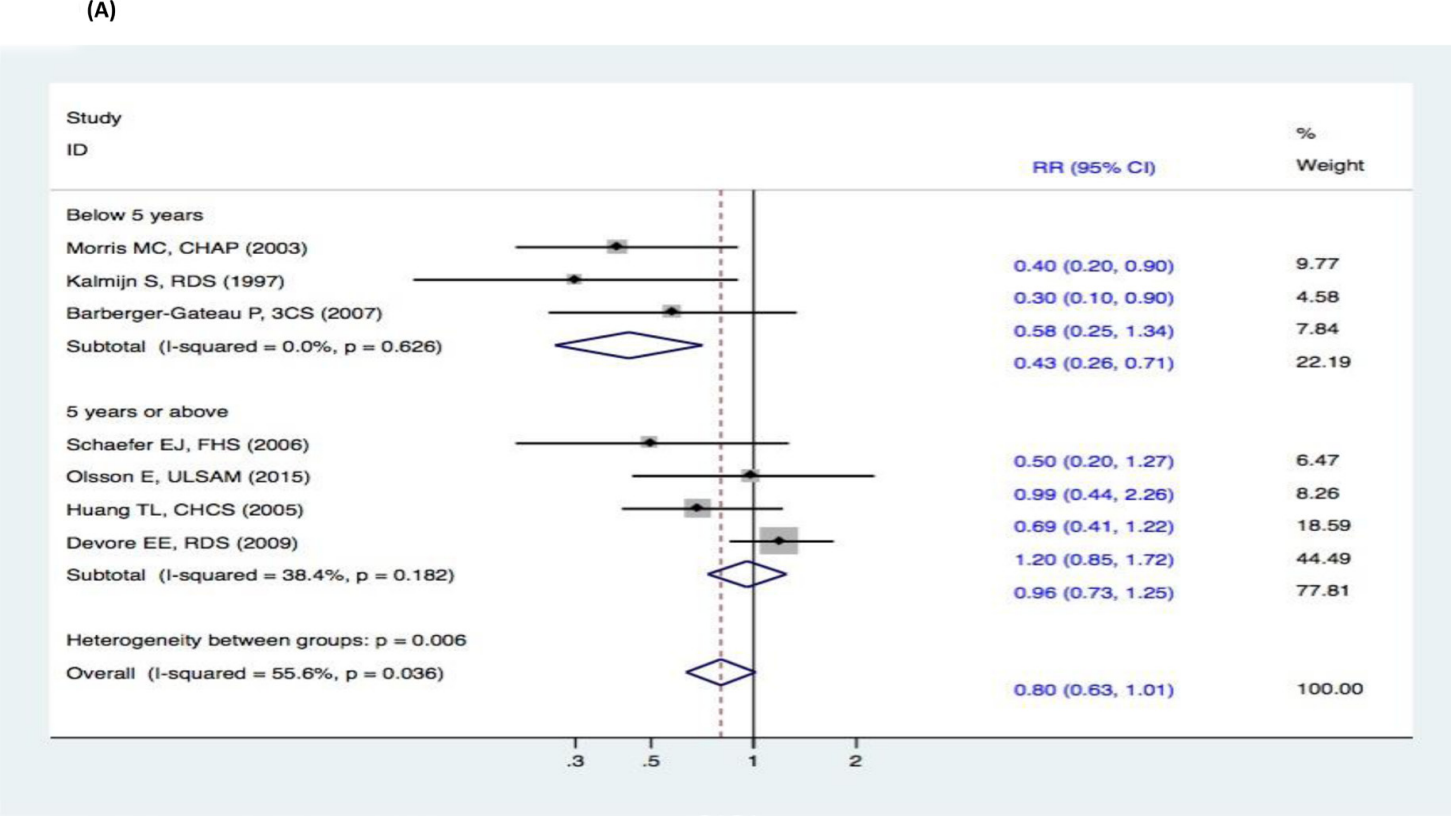

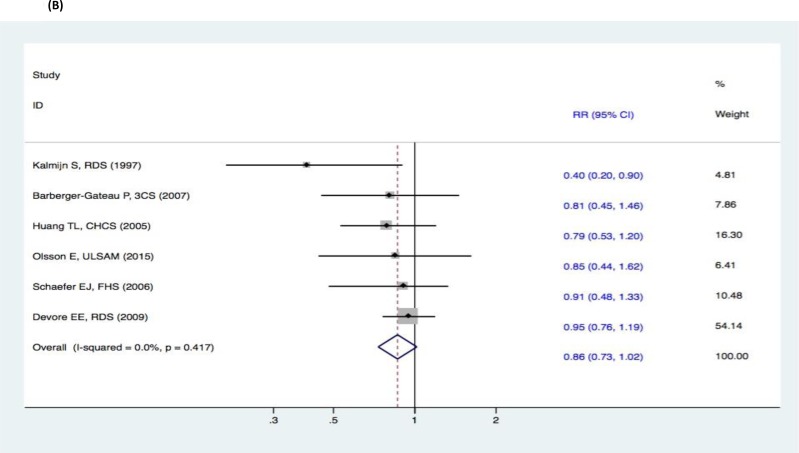

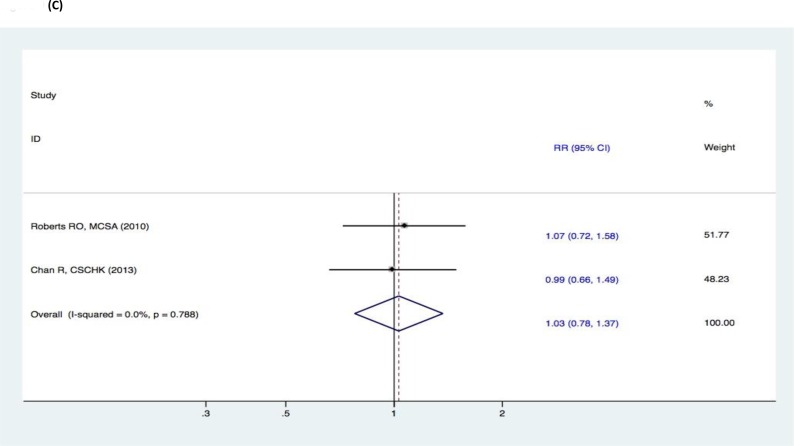
Subgroup analyses of mean follow-up duration for the correlation between fish consumption and risk of and risk of events: A. DAT; B. DAC; C. MCI Squares indicate study-specific risk estimates (size of the square reflects the study-specific statistical weight); horizontal lines indicate 95% CIs; diamond indicates the summary relative risk with its 95% CI. RR = relative risk; CI = confidence interval; DAT = dementia of Alzheimer type; DAC = dementia of all causes; MCI = mild cognitive impairment.

**Figure 10:**
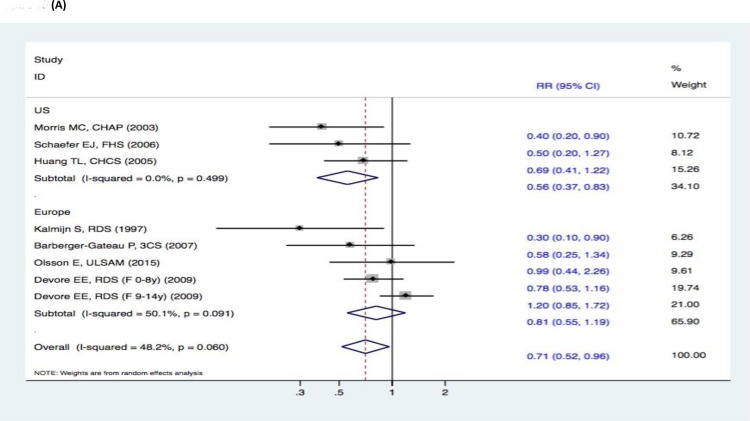

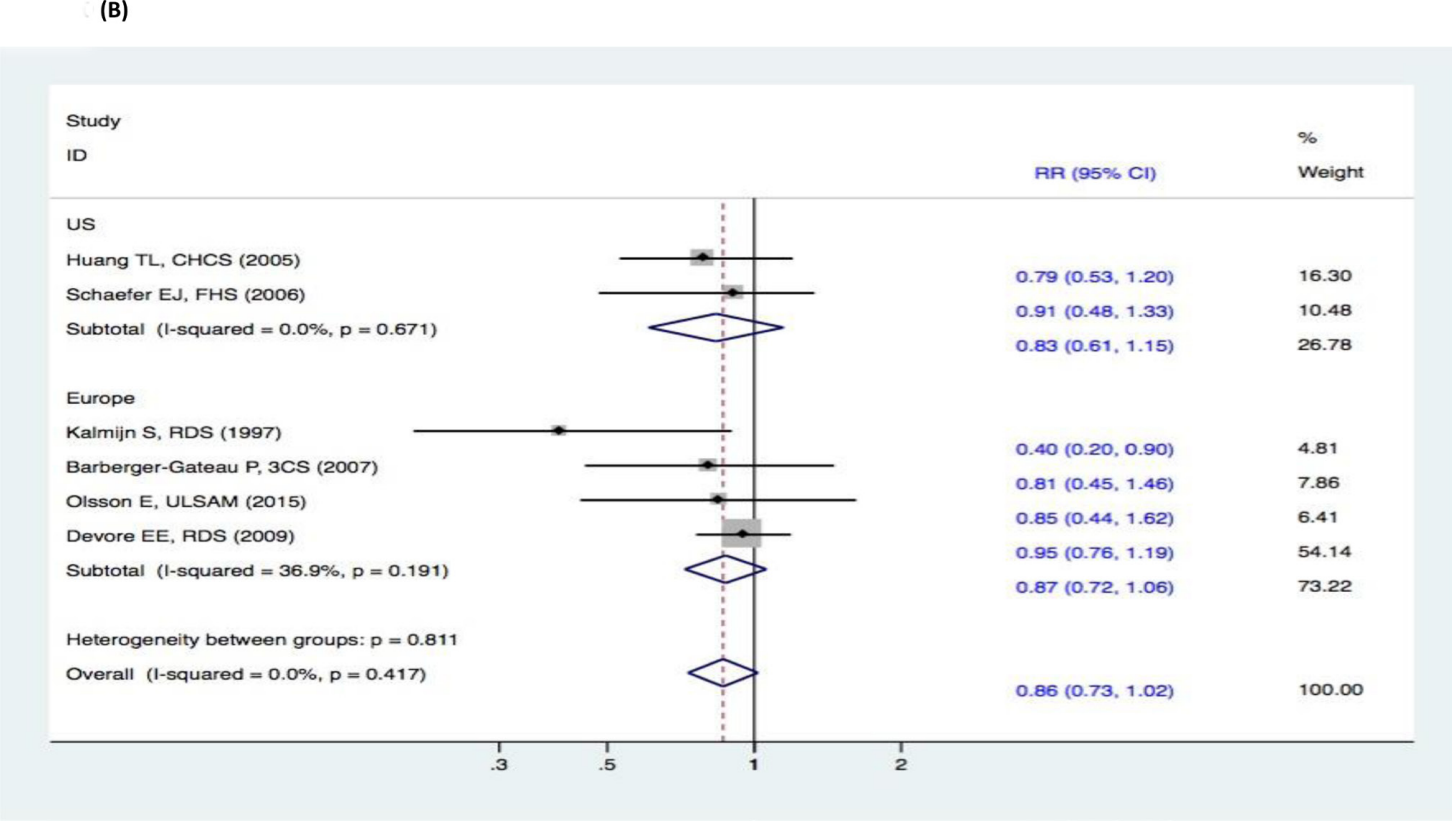

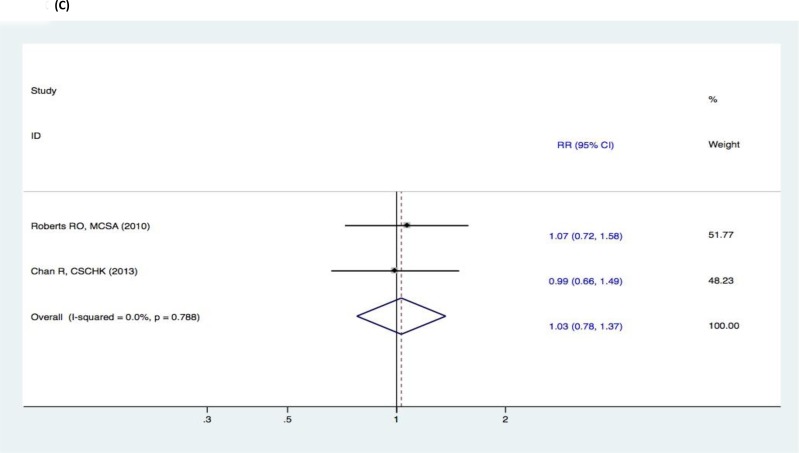
Subgroup analyses of geographic location for the correlation between fish consumption and risk of and risk of events: A. DAT; B. DAC; C. MCI Squares indicate study-specific risk estimates (size of the square reflects the study-specific statistical weight); horizontal lines indicate 95% CIs; diamond indicates the summary relative risk with its 95% CI. RR = relative risk; CI = confidence interval; DAT = dementia of Alzheimer type; DAC = dementia of all causes; MCI = mild cognitive impairment.

**Figure 11:**
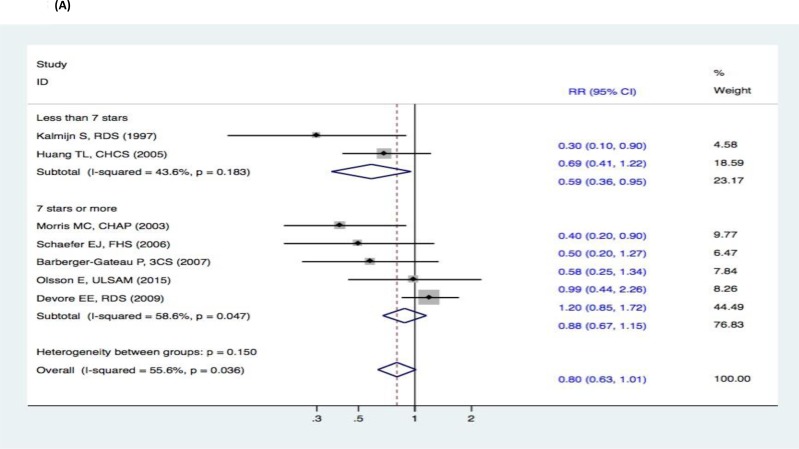

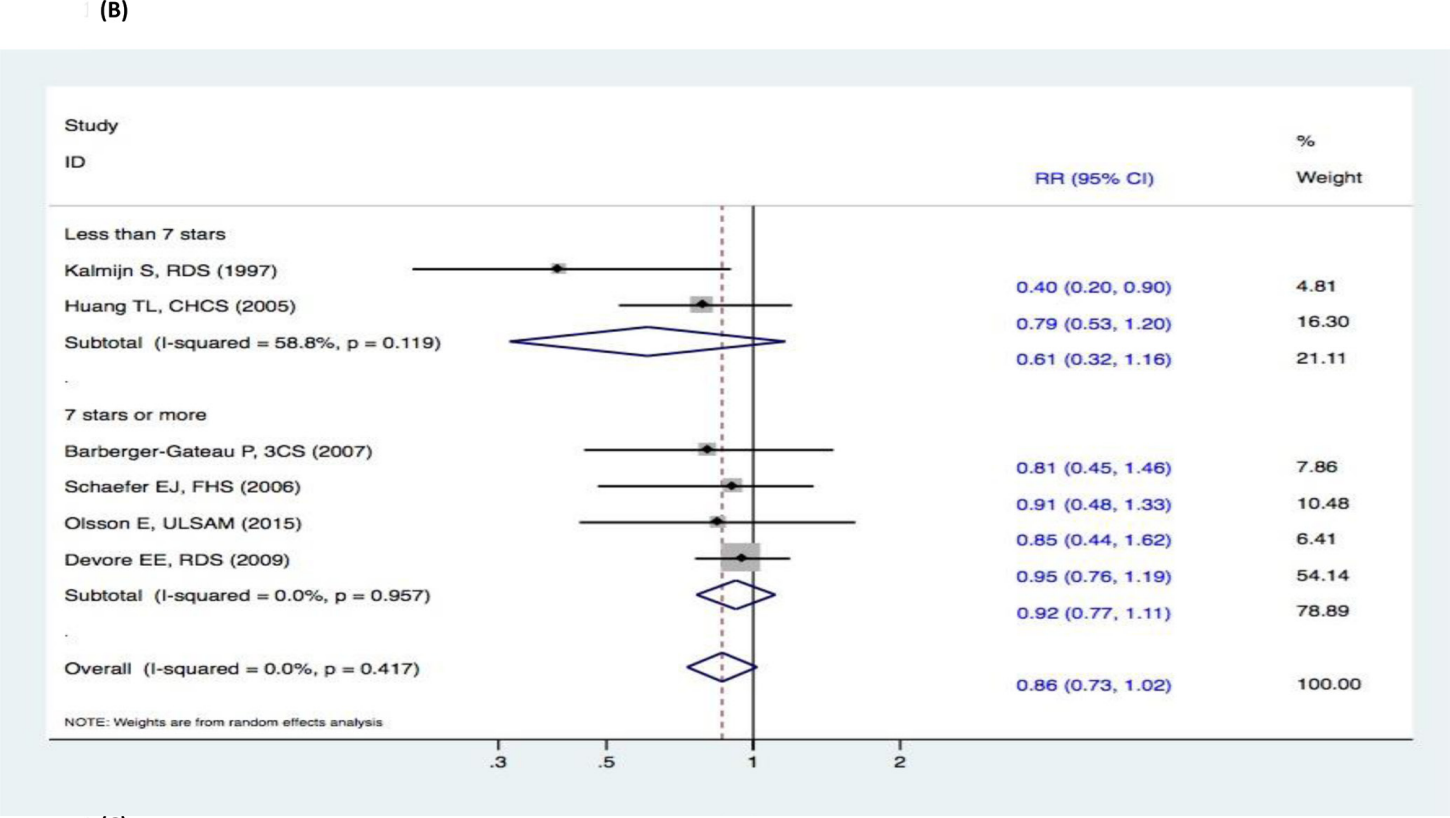

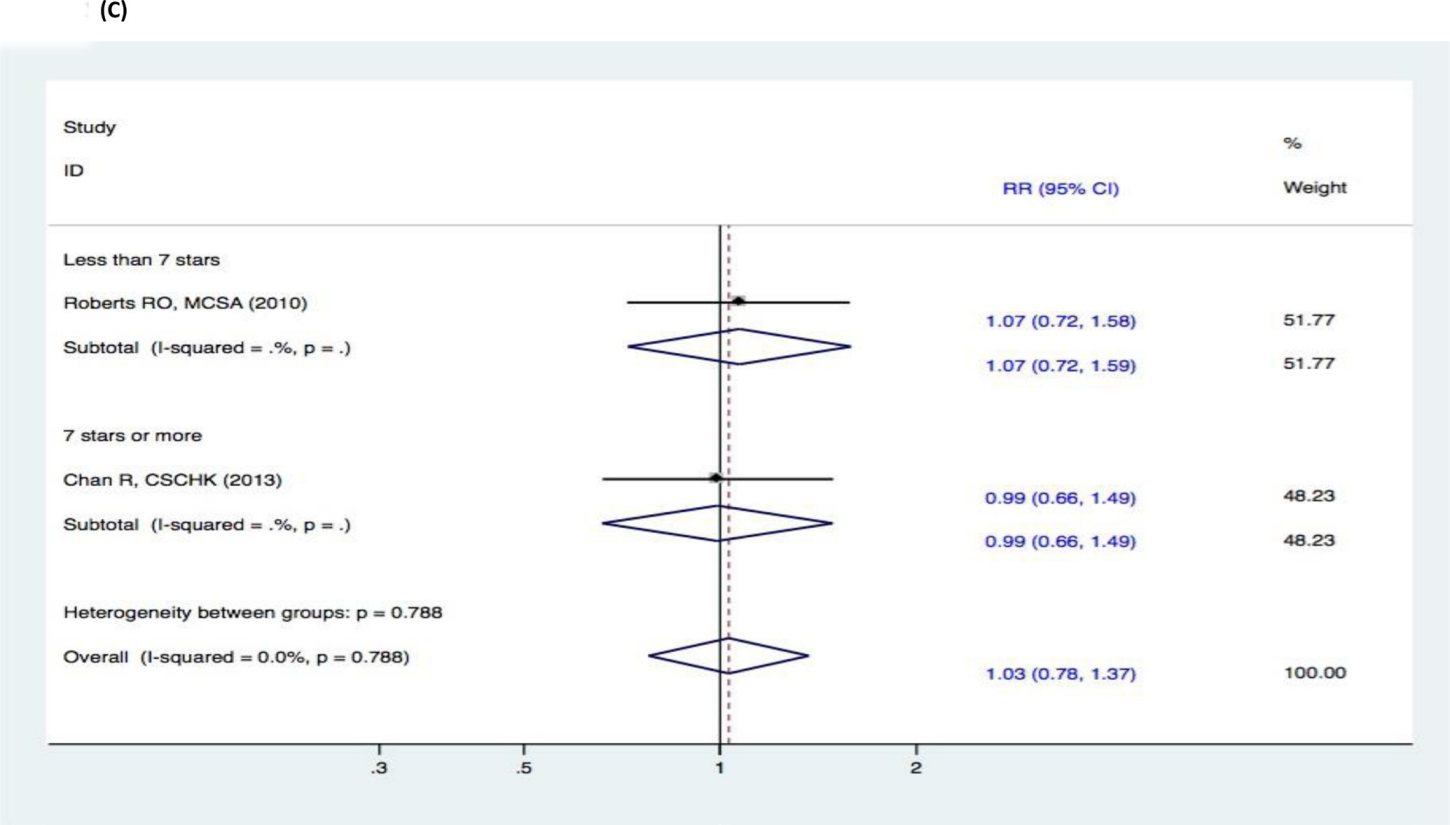
Subgroup analyses of study quality for the correlation between fish consumption and risk of and risk of events: A. DAT; B. DAC; C. MCI Squares indicate study-specific risk estimates (size of the square reflects the study-specific statistical weight); horizontal lines indicate 95% CIs; diamond indicates the summary relative risk with its 95% CI. RR = relative risk; CI = confidence interval; DAT = dementia of Alzheimer type; DAC = dementia of all causes; MCI = mild cognitive impairment.

There were no significant changes in the subgroup analyses of the association between fish intake and the risk of cognitive decline (DAT, DAC, and MCI) (Figure 9–11). Three studies [15–17] reported fish intake, short-term follow-up, and the risk of DAT. The synthesized evidence for the risk of DAT seemed much lower in the studies that followed up for less than 5 years (RR, 0.43 [95% CI, 0.26-0.71]). However, no protective effects were observed in the pooled results of four studies [18–21] with a follow-up of ≥5 years (RR, 0.96 [95% CI, 0.73-1.25]) that assessed the overall effects (RR, 0.80 [95% CI, 0.63-1.01]) of fish intake and the risk of DAT (Figure 9). Furthermore, seven studies [15–21] reported the effects of geographic location and fish intake on the risk of DAT. There was a reduced risk of DAT in the pooled results of three studies [15, 18, 20] conducted in the United States (RR, 0.56 [95% CI, 0.37-0.83]). However, there was no positive effect in the synthesized data from four studies [16–17, 19, 21] performed in Europe (RR, 0.81 [95% CI, 0.55-1.19]) (Figure 10). Although the pooled effects for the relative risks seemingly indicated statistical differences (RR, 0.71 [95% CI, 0.52-0.96]), this was probably a statistically significant association due to the type II error caused by the relatively small number of reports and wide confidence intervals. Additionally, the RR magnitude of DAT in two studies [16, 18] with low quality (less than 7 stars in the NOS scores) involving fish intake trended toward a lower risk of DAT (RR, 0.59 [95% CI, 0.36-0.95]). However, there were no statistically significant correlations in the pooled analysis of five high quality studies (7 stars or more in the NOS scores) [15, 17, 19, 20–21] (RR, 0.88 [95% CI, 0.67-1.15]) and overall effects (RR, 0.80 [95% CI, 0.63-1.01]) involving fish intake and the risk of DAT (Figure 11). Limited evidence of heterogeneity was found among studies or within subgroups.

### Meta-analysis based on dose-response data

**Figure 12:**
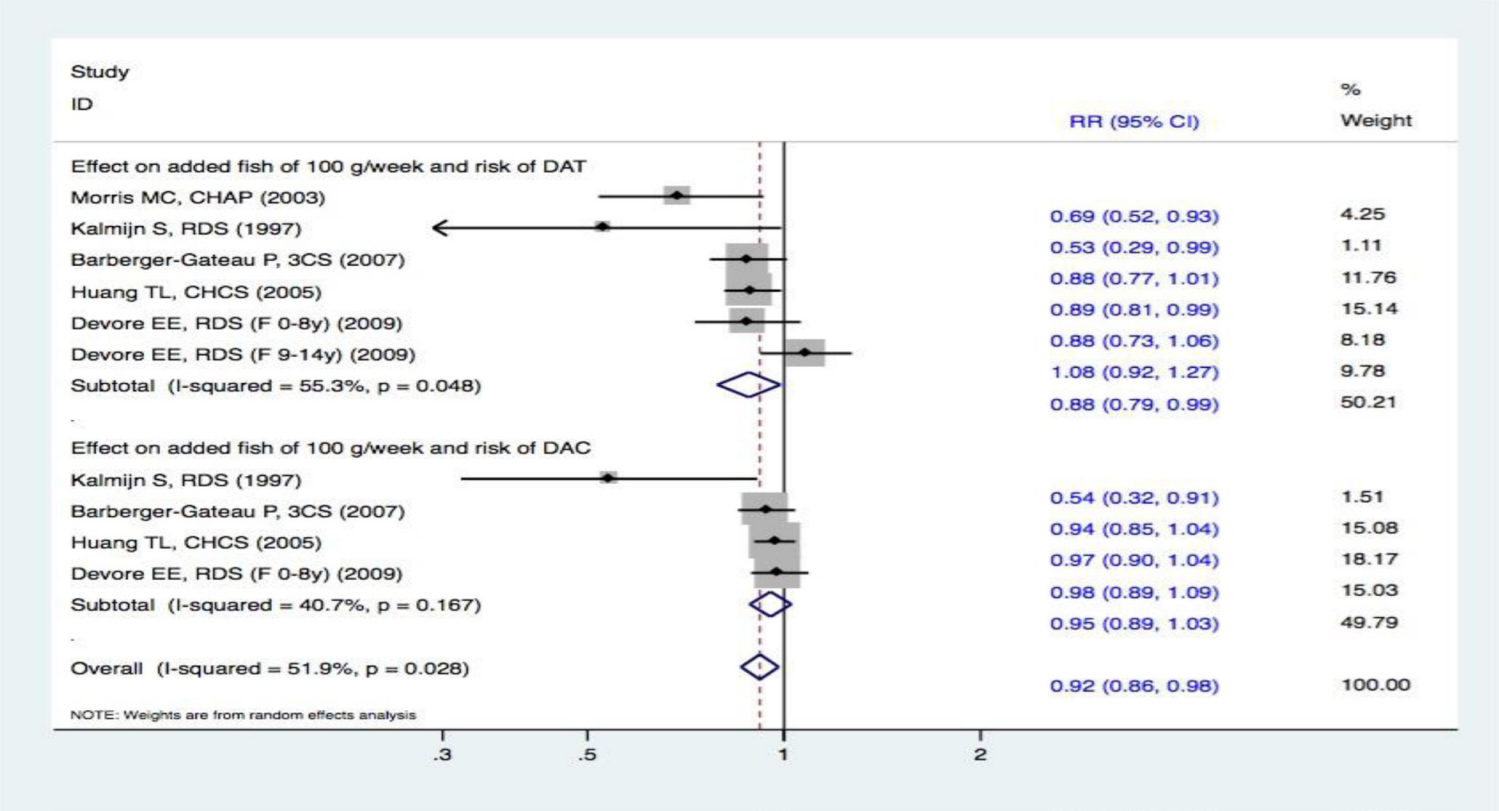
Meta-analysis based on dose-response data, i.e effect on added fish of 100 g/week and risk of events: **A**. DAT; **B**. DAC. Squares indicate study-specific risk estimates (size of the square reflects the study-specific statistical weight); horizontal lines indicate 95% CIs; diamond indicates the summary relative risk with its 95% CI. RR = relative risk; CI = confidence interval; DAT = dementia of Alzheimer type; DAC = dementia of all causes.

Dose-response meta-analyses were performed for fish intake and the risk of only DAT and DAC because we could not extract specific data for the other types of FDI. Studies that included only two categories of fish intake were excluded from further analysis because meta-analysis based on dose-response calculates require at least three categories of exposure involving the distribution of cases and person-time. For the dose-response synthesized evidence, three studies found a decreased risk of DAT with a 100-g/week increase in fish intake [15–16, 18]. In contrast, two studies [17, 19] failed to show such an association. Consequently, the pooled effect estimates demonstrated that an increased fish intake of 100 g/week was associated with an additional 12% reduced risk of DAT (RR, 0.88 [95% CI, 0.79-0.99]). In other words, the synthesized evidence found that increased fish intake significantly protected against the risk of DAT. Furthermore, one study [16] revealed that a 100-g/week increased fish intake reduced the risk of DAC, whereas three studies [17–19] failed to show a similar correlation. Thus, there was no statistically significant correlation in the pooled results (RR, 0.95 [95% CI, 0.89-1.03]). Therefore, increasing fish intake by 100 g/week had no obvious effect on the risk of DAC (Figure 12).

## DISCUSSION

### Summary of evidence

In this meta-analysis of nine studies, there was a significant association between fish intake and the risk of DAT when the highest and lowest categories of fish consumption were compared. Subjects with a higher intake of fish had a 20% (95% CI 3-35%) decreased risk of DAT, but there were no similar correlations with the risk of DAC and MCI. Moreover, the reduced risk of DAT, DAC, and MCI was not significantly associated with the intake of n-3 FAs, DHA, and EPA. Additionally, the synthesized dose-response evidence indicated that an increase in fish intake of 100 g/week reduced the risk of DAT by an additional 12% (RR, 0.88 [95% CI, 0.79-0.99]). Limited evidence of heterogeneity was found across studies or within subgroups. Overall, these analyses seemingly confirmed the inverse correlation between fish intake and the risk of DAT, whereby a higher consumption of fish could lower the risk of DAT. However, there was no statistical evidence for a link between n-3 FAs, DHA, or EPA and the risk of cognitive decline.

Due to limited methodological quality among the included studies, no definitive correlation could be verified between FDI and the risk of cognitive decline based on the present evidence. Thus, recommendations for specific decision-making should be interpreted with caution. Further strictly designed studies with specified quantification indices are needed to confirm these findings.

### Comparison of the findings with previous studies

Several studies assessing fish intake and the risk of dementia have been reported, including case reports, case series, controlled studies, and randomized controlled trials. However, no meta-analyses have focused on the correlations between a fish-oriented diet (fish, n-3 FAs, DHA, or EPA) and cognitive decline (e.g., DAT, DAC, or MCI). Thus, this is the first review to evaluate the role of FDI in the risk of cognitive disorders by searching the published literature.

Some underlying biochemical hypotheses or other biological mechanisms could play an important role in disease progression or pathogenesis. For example, the high intake of n-3 FAs from fish or fish-related sources could reduce the risk of cardiovascular disease; therefore, it could potentially decrease the risk of DAT or other types of dementia *via* vascular mechanisms [25]. Furthermore, DAT could also be influenced by a fish-oriented diet *via* other mechanisms. Insulin resistance was connected to a higher consumption of fish-related fats, whereas high concentrations of insulin might be associated with a high risk of DAT [26–27]. In addition, a high consumption of fats probably accelerated the oxidation of carbohydrate energy [28]; this process could contribute to cardiovascular diseases and result in DAT or other types of dementia.

Moreover, several findings showed a role for fish-related fat consumption in the pathogenesis of DAT. The deposition of amyloid-β (A-β) in animal brains could be induced by the consumption of a diet high in cholesterol [29], whereas the accumulation of neuronal A-β was increased in rabbits with hyperlipidemia [30]. Furthermore, reducing cholesterol concentrations using drugs [31] (e.g. 3 hydroxy-3methylglutaryl-coenzyme A (HMG-CoA) reductase inhibitors) led to a decreased risk of DAT. Additionally, apolipoprotein E (ApoE), a gene relevant to DAT, was closely associated with lipid metabolism [32], which could dual-directionally match the concentrations of cholesterol in lines with fish-related fats consumptions. In contrast, subjects with the ApoE-4 allele (indicating a higher risk of DAT) had high concentrations of cholesterol [33]. In contrast, subjects with the ApoE-2 allele (which might lower the risk of DAT) had relatively low concentrations of cholesterol [34].

Although a series of prospective studies reported correlations between a fish-oriented diet and the risk of DAT, the potential mechanisms behind these correlations were unclear. By following up 980 elderly subjects for 4 years, researchers demonstrated that the risk of DAT was highest in subjects carrying the ApoE-4 allele in the highest quartile of total fat consumption [35]. Another study found that the high consumption of saturated and trans unsaturated FAs was related to an increased risk of DAT regardless of the ApoE genotype [36]. In addition, the intake of fish-oriented fats was correlated with a reduced risk of DAT [37]. Nevertheless, no type of fat consumption was correlated with DAT or other types of dementia in a study performed in over 5000 subjects aged ≥55 years [38]. In summary, inconsistencies focusing on the consumption of a fish-oriented diet and the risk of cognitive decline were found, and specific recommendations could not be made based on the recent studies. However, a diet high in fish-related fats was correlated with a reduced risk of vascular disease, which might be of significance. Therefore, it might be sensible to take these potential benefits into account for the further management of cognitive decline.

### Clinical implications

A fish-oriented diet could have additive benefits for subjects with DAT since there is a potential link between FDI and cognitive decline. However, several caveats in the evidence linking diet to cognitive declines were found in the current study. The first key point focuses on the improved demand for the quantification of nutrients. Most nutrients were measured using dietary questionnaires containing relative terms rather than any absolute measurements. In other words, more or less of a nutrient could be inferred to be linked with better or worse outcomes. However, the description of the actual quantities and intervals of nutrient consumption in relevant observational studies was insufficient to allow any specific inferences to be drawn. Moreover, the exposures were selected by the subjects (not the investigators), which might have contributed to a series of undetected confounders. The above confounding issues and potential bias might be largely resolved by specific trial design in RCTs, which are viewed as the ideal approach to study the effects of diet on disease risk. However, due to the long periods of preclinical DAT or other type of dementia, it is impossible for researchers to conduct trials based on primary prevention using diet. Furthermore, the development of DAT might be the results of a subjects' life-long exposures or timing of positive events starting in later life. However, the latency duration in subjects with DAT (i.e., the amount by which dietary interventions postponed or prevented disease progression) could be several decades, which is unclear based on recent evidence. Since studies assessing the correlation between diet and DAT were performed in the subjects aged > 65 years, a potential advanced phase involving the latency duration of DAT was seemingly observed in these aged groups. Consequently, evidence regarding the effects of dietary interventions on the course of different diseases is limited. Additionally, the different diets in included studies might affect the condition of the preclinical DAT, and the final exposure results also could be biased by preclinical cognitive-related conditions. Together, these factors likely led to incorrect interpretation or bias.

Another important consideration is the methodological formulation. Recently, most subjects have been diagnosed based on routine guidelines for dementia such as the diagnostic and statistical manual of mental disorders fourth edition (DSM-IV). However, the detections of similar data did not always indicate qualified results. We increasingly considered the present guidelines do not guarantee consistency with the actual integrity of subjects' cognitive declines. Thus, the National Institutes of Mental Health has attempted to define some criteria for the provisional diagnosis of cognitive decline. In addition, dozens of scales have been formulated to evaluate the accompanying disorders in subjects with cognitive decline for the purpose of better estimating the effects of or tracing the progression of disease conditions. For the specific assessment of subjects with cognitive decline, the patterns of cognitive decline should be further updated using similar rating scores and structured interview approaches, especially due to the measurement of potential patients with severe cognitive damages using initial self-reported procedures. In clinical practice, symptoms could be under-reported by patients or over-reported by caregivers, or biased reports could be caused by stress or other emotional factors regarding the subjects themselves. Therefore, clinical researchers should take some preventive measures to balance and evaluate the sources of information.

### Limitations

This meta-analysis provides information regarding the association between FDI and the risk of cognitive decline. However, there are several limitations. First, the evidence in this meta-analysis was derived from previously published observational studies. However, it is possible that some eligible documents were missed in the initial searches, thereby creating potential bias. Second, although measures were taken to control for underlying confounders in most of the included studies, some remaining confounders might still exist. Third, no unifying standards were used to define the time of detection of the cognitive decline among the included studies. Finally, the relatively limited sample size of the included studies, and evidence for interpretation and decision-making are needed for further study.

## CONCLUSIONS

In conclusion, the findings of this study confirmed an inverse correlation between fish intake and the risk of DAT. Therefore, a higher consumption of fish was strongly associated with a lower risk of DAT. However, there was no statistical link between n-3 FAs, DHA, or EPA intake and the risk of cognitive decline. More prospective studies that specifically quantify FDI will help the more accurate assessment of different intakes of a fish-oriented diet.

## MATERIALS AND METHODS

The guidelines of the Meta-analysis of Observational Studies in Epidemiology Group (MOOSE) [39], the statement of Preferred Reporting Items for Systematic Reviews and Meta-analyses (PRISMA) [40], and the statement of Strengthening the Reporting of Observational Studies in Epidemiology (STROBE) [41] were strictly followed and adhered to throughout this study.

### Literature search

Cohort studies with a prospective design that investigated cognitive decline and included data on the exposure to FDI were included in this review. Two researchers performed systemic searches to identify potential studies published in December 2016 and earlier using PubMed, Embase, and Web of Science. The combined searches focused on MeSH terms and free-text retrieval using the following search terms: “diet” or “dietary” or “fish” or “omega-3 fatty acids” and “cognitive impairment” or “cognitive decline” or “cognitive damage” or “Alzheimer's disease” or “dementia.” Furthermore, the reference lists of all retrieved articles were manually checked for potentially relevant citations. The results of the final literature reviews were updated on December 30th, 2016.

### Criteria for inclusion

Publications qualified for inclusion in this meta-analysis if they matched the following specific criteria: i) prospective cohort studies; ii) reported the potential exposure of FDI (including fish, n-3 FAs supplementation, or fish-related products.); iii) follow-up for more than 1 year in a general population with a high risk of cognitive decline (e.g., populations aged ≥55 years); iv) the outcome measures included cognitive decline (including DAT, MCI, and DAC); and v) the estimated relative risks (RRs) of cognitive decline were compared between the highest and lowest categories of FDI, and 95% confidence intervals (CIs) or other indices that could be inferred were provided. Meanwhile, the exclusion criteria were as follows: a) previous records of mechanistic descriptive reports, review articles, and animal experiments; b) the lack of concrete data describing cognitive decline; c) studies published without full text.

### Study identification

In addition to the searches conducted using electronic databases, the reference lists of identified documents were hand-searched for identify further potential studies. Furthermore, we attempted to contact potential manufacturers of anti-dementia drugs or other experts involved in cognitive decline research. Initially, a researcher scanned the titles and abstracts of the publications. Then, the potentially eligible studies were read in full by two independent researchers. Disagreements were resolved by consulting a third or fourth researcher or reaching a consensus. Overall, two researchers agreed on > 90% of the studies for inclusion and exclusion. Finally, a manual retrieval was performed for all the correlated and reviewed documents, catalogs, and bibliographies of regular articles, as well as the abstracts of meetings held by the National Institute on Aging and Alzheimer's Association (NIA-AA), Risk Evaluation and Education for Alzheimer's Disease (REVEAL), Alzheimer's Disease Neuroimaging Initiative (ADNI), Anti-amyloid Treatment in Asymptomatic Alzheimer's Disease (A-4 Study), Alzheimer's Association International Conference (AAIC), Alzheimer's Disease International (ADI), or other dementia associations.

### Quality assessment and data extraction

The quality of the eligible studies was assessed using the Newcastle-Ottawa Scale (NOS) for non-randomized controlled trials. The specific criteria used for the estimates are shown in Supplemental Table 1. A “star system” was used to assess every included prospective cohort study using the following three broad domains [24]: i) the items for study selection; ii) the items for comparisons between groups; and iii) the items for outcome assessments. The total scores ranged from 1-9 stars according to the literature quality, and studies with seven or more stars are viewed as high quality.

Two independent researchers used standardized literature collection forms for study recruitment and data extraction. The abstracted items consisted of the authors' names, the year of literature publication, the study population, the patient characteristics (e.g., the sample size at baseline and follow-up, gender, age, medications and therapies, diagnoses, the methods used to measure cognitive declines, the exclusion criteria at baseline, the duration of follow-up, and withdrawals), endpoint estimates (disease definitions and approaches used for disease detection), the disease incidence rates, and the numbers of positive cases in each group. A third researcher was used to solve any discrepancies and achieve a consensus.

### Statistical analysis and data synthesis

The meta-analysis of risk calculates of cognitive decline were performed to compare the highest and lowest exposures to FDI. Since fish consumption was the vital source of n-3 FAs but not the final form of n-3 FAs intake, the data regarding fish and other dietary intake of FDI (i.e., n-3 FAs, DHA, or EPA) were pooled respectively. Dose-response meta-analyses of FDI and the risk of cognitive decline were also conducted using approaches reported previously [42–44]. These analyses provided the estimates of a pooled relative risk across studies with a common comparison unit to calculate possible linear dose-response associations. For citations that described fish consumption in “servings,” one serving was assumed to be 100 g fish [45–46]. The relative risk for fish intake with an increase of 100 g/week was then estimated for each potential study and the final data synthesis were handled together.

A fixed-effects model (FEM) was applied to calculate pooled relative risks (RRs) and 95% confidence intervals (CIs) if no evidence of heterogeneity was observed. Otherwise, a random-effects model (REM) was used for assessments. Chi-square tests and I-squared (I^2^) statistics were adopted to explore potential heterogeneity among studies. Funnel plots were used to inspect possible publication bias. Subgroup analyses were conducted on FDI and the risk of cognitive decline based on the period of follow-up, geographic region, and study quality to assess the potential impact factors. All analyses were performed using Stata SE, version 14.1 (Stata Corp, College Station, TX, USA).

## SUPPLEMENTARY MATERIALS TABLES



## References

[1] Alzheimer's Association (2016). Alzheimer's disease facts and figures. Alzheimers Dement.

[2] Collins PY, Patel V, Joestl SS, March D, Insel TR, Daar AS, Anderson W, Dhansay MA, Phillips A, Shurin S, Walport M, Ewart W, Scientific Advisory Board and the Executive Committee of the Grand Challenges on Global Mental Health (2011). Grand challenges in global mental health. Nature.

[3] Morris MC (2016). Nutrition and risk of dementia: overview and methodological issues. Ann N Y Acad Sci.

[4] Karr JE, Alexander JE, Winningham RG (2011). Omega-3 polyunsaturated fatty acids and cognition throughout the lifespan: a review. Nutr Neurosci.

[5] Féart C, Samieri C, Rondeau V, Amieva H, Portet F, Dartigues JF, Scarmeas N, Barberger-Gateau P (2009). Adherence to a Mediterranean diet, cognitive decline, and risk of dementia. JAMA.

[6] Weiser MJ, Butt CM, Mohajeri MH (2016). Docosahexaenoic Acid and Cognition throughout the Lifespan. Nutrients.

[7] Kou W, Luchtman D, Song C (2008). Eicosapentaenoic acid (EPA) increases cell viability and expression of neurotrophin receptors in retinoic acid and brain-derived neurotrophic factor differentiated SH-SY5Y cells. Eur J Nutr.

[8] Lim SY, Suzuki H (2000). Intakes of dietary docosahexaenoic acid ethyl ester and egg phosphatidylcholine improve maze-learning ability in young and old mice. J Nutr.

[9] Calon F, Cole G (2007). Neuroprotective action of omega-3 polyunsaturated fatty acids against neurodegenerative diseases: evidence from animal studies. Prostaglandins Leukot Essent Fatty Acids.

[10] Hu FB, Bronner L, Willett WC, Stampfer MJ, Rexrode KM, Albert CM, Hunter D, Manson JE (2002). Fish and omega-3 fatty acid intake and risk of coronary heart disease in women. JAMA.

[11] He K, Song Y, Daviglus ML, Liu K, Van_Horn L, Dyer AR, Greenland P (2004). Accumulated evidence on fish consumption and coronary heart disease mortality: a meta-analysis of cohort studies. Circulation.

[12] Daviglus ML, Stamler J, Orencia AJ, Dyer AR, Liu K, Greenland P, Walsh MK, Morris D, Shekelle RB (1997). Fish consumption and the 30-year risk of fatal myocardial infarction. N Engl J Med.

[13] Kromhout D, Bosschieter EB, de_Lezenne Coulander C (1985). The inverse relation between fish consumption and 20-year mortality from coronary heart disease. N Engl J Med.

[14] He K, Rimm EB, Merchant A, Rosner BA, Stampfer MJ, Willett WC, Ascherio A (2002). Fish consumption and risk of stroke in men. JAMA.

[15] Morris MC, Evans DA, Bienias JL, Tangney CC, Bennett DA, Wilson RS, Aggarwal N, Schneider J (2003). Consumption of fish and n-3 fatty acids and risk of incident Alzheimer disease. Arch Neurol.

[16] Kalmijn S, Launer LJ, Ott A, Witteman JC, Hofman A, Breteler MM (1997). Dietary fat intake and the risk of incident dementia in the Rotterdam Study. Ann Neurol.

[17] Barberger-Gateau P, Raffaitin C, Letenneur L, Berr C, Tzourio C, Dartigues JF, Alpérovitch A (2007). Dietary patterns and risk of dementia: the Three-City cohort study. Neurology.

[18] Huang TL, Zandi PP, Tucker KL, Fitzpatrick AL, Kuller LH, Fried LP, Burke GL, Carlson MC (2005). Benefits of fatty fish on dementia risk are stronger for those without APOE epsilon4. Neurology.

[19] Devore EE, Grodstein F, van_Rooij FJ, Hofman A, Rosner B, Stampfer MJ, Witteman JC, Breteler MM (2009). Dietary intake of fish and omega-3 fatty acids in relation to long-term dementia risk. Am J Clin Nutr.

[20] Schaefer EJ, Bongard V, Beiser AS, Lamon-Fava S, Robins SJ, Au R, Tucker KL, Kyle DJ, Wilson PW, Wolf PA (2006). Plasma phosphatidylcholine docosahexaenoic acid content and risk of dementia and Alzheimer disease: the Framingham Heart Study. Arch Neurol.

[21] Olsson E, Karlström B, Kilander L, Byberg L, Cederholm T, Sjögren P (2015). Dietary patterns and cognitive dysfunction in a 12-year follow-up study of 70 year old men. J Alzheimers Dis.

[22] Roberts RO, Cerhan JR, Geda YE, Knopman DS, Cha RH, Christianson TJ, Pankratz VS, Ivnik RJ, O'Connor HM, Petersen RC (2010). Polyunsaturated fatty acids and reduced odds of MCI: the Mayo Clinic Study of Aging. J Alzheimers Dis.

[23] Chan R, Chan D, Woo J (2013). A cross sectional study to examine the association between dietary patterns and cognitive impairment in older Chinese people in Hong Kong. J Nutr Health Aging.

[24] Wells GA, Shea BJ, O'Connell D, Peterson J, Welch V, Losos M (2000). The Newcastle-Ottawa Scale (NOS) for assessing the quality of nonrandomisedstudies in meta-analyses. 3rd Symposium on Systematic Reviews: Beyondthe Basics.

[25] Hu FB, Willett WC (2002). Optimal diets for prevention of coronary heart disease. JAMA.

[26] Sears B, Perry M (2015). The role of fatty acids in insulin resistance. Lipids Health Dis.

[27] Bray GA, Lovejoy JC, Smith SR, DeLany JP, Lefevre M, Hwang D, Ryan DH, York DA (2002). The influence of different fats and fatty acids on obesity, insulin resistance and inflammation. J Nutr.

[28] Cohn JS (2002). Oxidized fat in the diet, postprandial lipaemia and cardiovascular disease. Curr Opin Lipidol.

[29] Hardy JA, Higgins GA (1992). Alzheimer's disease: the amyloid cascade hypothesis. Science.

[30] Sparks DL, Martins R, Martin T (2002). Cholesterol and cognition: rationale for the AD cholesterol-lowering treatment trial and sex-related Differences in beta-amyloid accumulation in the brains of spontaneously hypercholesterolemic Watanabe rabbits. Ann N Y Acad Sci.

[31] Jick H, Zornberg GL, Jick SS, Seshadri S, Drachman DA (2000). Statins and the risk of dementia. Lancet.

[32] Selkoe DJ (1997). Alzheimer's disease: genotypes, phenotypes, and treatments. Science.

[33] Petot GJ, Traore F, Debanne SM, Lerner AJ, Smyth KA, Friedland RP (2003). Interactions of apolipoprotein E genotype and dietary fat intake of healthy older persons during mid-adult life. Metabolism.

[34] Erkkilä AT, Sarkkinen ES, Lindi V, Lehto S, Laakso M, Uusitupa MI (2001). APOE polymorphism and the hypertriglyceridemic effect of dietary sucrose. Am J Clin Nutr.

[35] Luchsinger JA, Tang MX, Shea S, Mayeux R (2002). Caloric intake and the risk of Alzheimer disease. Arch Neurol.

[36] Morris MC, Evans DA, Bienias JL, Tangney CC, Bennett DA, Aggarwal N, Schneider J, Wilson RS (2003). Dietary fats and the risk of incident Alzheimer disease. Arch Neurol.

[37] Morris MC, Evans DA, Bienias JL, Tangney CC, Wilson RS (2004). Dietary fat intake and 6-year cognitive change in an older biracial community population. Neurology.

[38] Engelhart MJ, Geerlings MI, Ruitenberg A, Van_Swieten JC, Hofman A, Witteman JC, Breteler MM (2002). Diet and risk of dementia: Does fat matter?. The Rotterdam Study. Neurology.

[39] Stroup DF, Berlin JA, Morton SC, Olkin I, Williamson GD, Rennie D, Moher D, Becker BJ, Sipe TA, Thacker SB (2000). Meta-analysis of observational studies in epidemiology: a proposal for reporting. Meta-analysis Of Observational Studies in Epidemiology (MOOSE) group. JAMA.

[40] Moher D, Liberati A, Tetzlaff J, Altman DG, PRISMA Group (2009). Preferred reporting items for systematic reviews and meta-analyses: the PRISMA statement. BMJ.

[41] von_Elm E, Altman DG, Egger M, Pocock SJ, Gøtzsche PC, Vandenbroucke JP (2007). The Strengthening the Reporting of Observational Studies in Epidemiology (STROBE) statement: guidelines for reporting observational studies. Lancet.

[42] Greenland S, Longnecker MP (1992). Methods for trend estimation from summarized dose-response data, with applications to meta-analysis. Am J Epidemiol.

[43] Larsson SC, Orsini N (2011). Fish consumption and the risk of stroke: a dose-response meta-analysis. Stroke.

[44] Jiang L, Hou R, Gong TT, Wu QJ (2015). Dietary fat intake and endometrial cancer risk: dose-response meta-analysis of epidemiological studies. Sci Rep.

[45] Guevel MR, Sirot V, Volatier JL, Leblanc JC (2008). A risk-benefit analysis of French high fish consumption: a QALY approach. Risk Anal.

[46] Bouzan C, Cohen JT, Connor WE, Kris-Etherton PM, Gray GM, König A, Lawrence RS, Savitz DA, Teutsch SM (2005). A quantitative analysis of fish consumption and stroke risk. Am J Prev Med.

