# Supplementary material for: An exploration of the role of a fish-oriented diet in cognitive decline: a systematic review of the literature
Source: Oncotarget. 2017 Mar 17;8(24):39877–95. doi: 10.18632/oncotarget.16347 (PMC5503660; doi:10.18632/oncotarget.16347)
Supplement: Supplementary file 1 [file oncotarget-08-39877-s001.docx]

**Appendix**

**Supplemental Tables 1. Newcastle-Ottawa Quality Assessment Scale: Cohort Studies**

| **Variable** | **Possible Response** |
| --- | --- |
| **1. Selection** |  |
| 1.1 Representativeness of the exposed cohort | a) Truly representative of the average ________ (describe) in the community*  b) Somewhat representative of the average _______ in the community*****  c) Selected group of users e.g. nurses, volunteers  d) No description of the derivation of the cohort |
| 1.2 Selection of the non-exposed cohort | a) Drawn from the same community as the exposed cohort*  b) Drawn from a different source  c) No description of the derivation of the non-exposed cohort |
| 1.3 Ascertainment of exposure | a) Secure record (eg surgical records) *****  b) Structured interview *****  c) Written self report  d) No description |
| 1.4 Demonstration that outcome of interest was not present at start of study | a) Yes*****  b) No |
| **2. Comparability** |  |
| Comparability of cohorts on the basis of the design or analysis | a) Study controls for _____ (select the most important factor) *****  b) Study controls for any additional factor (This criteria could be modified to indicate specific control for a second important factor.) ***** |
| **3. Outcome** |  |
| 3.1 Assessment of outcome | a) Independent blind assessment*****  b) Record linkage *****  c) Self report  d) No description |
| 3.2 Was follow-up long enough for outcomes to occur | a) Yes (select an adequate follow up period for outcome of interest) *****  b) No |
| 3.3 Adequacy of follow up of cohorts | a) Complete follow up - all subjects accounted for *****  b) Subjects lost to follow up unlikely to introduce bias - small number lost->__% (select an adequate%) follow up, or description provided of those lost) *****  c) Follow up rate <__% (select an adequate %) and no description of those lost  d) No statement |

**Note:** A study can be awarded a maximum of one star for each numbered item within the Selection and Outcome categories. A maximum of two stars can be given for Comparability.
